# Treatment-Resistant Nausea and Vomiting: A Challenge in Clinical Practice

**DOI:** 10.34172/mejdd.2025.437

**Published:** 2025-07-30

**Authors:** Roya Rafaiee, Raheleh Rafaiee, Alireza Bakhshipour

**Affiliations:** ^1^Department of Internal Medicine, School of Medicine, Zahedan University of Medical Sciences, Zahedan, Iran; ^2^Psychiatry and Behavioral Sciences Research Center, Addiction Institute, Mazandaran University of Medical Sciences, Sari, Iran; ^3^Infectious Diseases and Tropical Medicine Research Center, Resistant Tuberculosis Institute, Zahedan University of Medical Sciences, Zahedan, Iran

## Dear Editor,

 Treatment-resistant nausea and vomiting pose a major challenge in the clinical setting. Traditional pharmacological interventions are often ineffective, so more investigations are required. The medical literature suggests that suppression of the hypothalamic-pituitary-adrenal (HPA) axis by glucocorticoids, such as dexamethasone, can contribute significantly to treatment-resistant nausea and vomiting.^1^ This phenomenon is complex and involves several key mechanisms. Glucocorticoids can cause suppression of the HPA axis, resulting in decreased cortisol levels, which can impair the body’s stress response and exacerbate symptoms of nausea and vomiting.^2^ Cortisol suppression can disrupt the balance of neuroendocrine signals that regulate nausea, potentially leading to anticipatory nausea in patients.^3^

 Dexamethasone has been shown to induce resistance to chemotherapy in several types of cancer, including ovarian cancer, by upregulating survival genes that inhibit apoptosis, making treatment outcomes more difficult.^4^ This resistance can manifest as persistent nausea and vomiting as the body’s ability to manage these symptoms through normal physiological pathways is impaired.^3^ While glucocorticoids are commonly used to alleviate nausea, their role in suppressing the HPA axis and subsequent treatment resistance highlights a complex interplay that may require careful management strategies to avoid exacerbation of these symptoms.

 The use of supraphysiological doses of corticosteroids for more than 3 weeks has significant effects on the HPA axis, primarily through negative feedback mechanisms that suppress adrenal function.^5^ High-dose corticosteroids lead to a significant reduction in plasma cortisol levels, as shown in studies in which treatment with medroxyprogesterone acetate (MPA) led to a 76% decrease in cortisol concentrations.^6^ When treated with high-dose glucocorticoids, a significant attenuation of the responsiveness of the HPA axis was observed, with cortisol responses remaining suppressed for several days after treatment.^7^ Studies suggest that the HPA axis may recover within weeks of discontinuation of treatment, indicating transient suppression.^8^ Continuous administration of corticosteroids can lead to chronic suppression of the adrenal glands, which may not recover spontaneously after cessation of treatment, posing a risk during stressful events.^9^

 Adrenaline insufficiency, especially during stress or illness, can lead to severe symptoms such as nausea and vomiting that defy standard treatment. This condition results from inadequate production of cortisol, which is crucial for coping with stress responses.^10^ Under physiological stress conditions, such as in critically ill patients, the adrenal glands cannot produce enough cortisol, which exacerbates symptoms such as nausea and vomiting.^10^ Glucocorticoid replacement therapies often fail to mimic the natural cortisol rhythm, resulting in inadequate stress response and persistent symptoms.^11^ An increased need for cortisol during acute stress, e.g. during infections or operations, can also mean that glucocorticoid substitution is not sufficient.^12^

 Persistent nausea and vomiting due to adrenaline insufficiency are caused by the interaction of neurophysiological mechanisms and hormonal reactions. The emetic reflex is primarily coordinated by a network in the brainstem, including the area postrema and the nucleus of the tractus solitaire, which are activated by various stimuli, including hormonal changes due to adrenaline insufficiency.^3^ Catecholamines, particularly via alpha-adrenergic signaling pathways, have been shown to induce vomiting when administered centrally, suggesting their role in the emetic response.^13^ Adrenaline release is regulated by the hypothalamus, which responds to glucose insufficiency and associates metabolic states with nausea and vomiting.^14^ The disruption of gastric slow waves during nausea is modulated by both cholinergic and adrenergic signaling pathways, suggesting a complex interaction between these systems in response to adrenaline levels.^15^

 It should be noted that treatment-resistant nausea and vomiting may be associated with autoimmune diseases, particularly neuromyelitis optica (NMO) and its spectrum disorders. Corticosteroids have been shown to be very effective in managing these symptoms by reducing inflammation and modulating the immune response. NMO can be associated with intractable nausea and vomiting due to inflammation in the area postrema where autoantibodies target the aquaporin-4 channels.^16-18^ NMO can also manifest as a complication of systemic lupus erythematosus (SLE), where nausea and vomiting are the first symptoms, emphasizing the autoimmune nature of these clinical pictures. The use of corticosteroids is a standard approach in the treatment of autoimmune diseases and often leads to a rapid improvement in symptoms.^19^ In conclusion, in the management of patients with treatment-resistant nausea and vomiting, it is crucial to inquire about previous corticosteroid use, even if it was years ago. This is due to the long-lasting effects that corticosteroids can have on the body, particularly in terms of hormonal and immunological responses. A retrospective study has shown that many patients taking corticosteroids are not adequately monitored for adverse effects, indicating a gap in clinical practice.^20^ Corticosteroids can cause resistance in the body’s hormonal responses, as evidenced by studies linking aging and cortisol resistance to dexamethasone suppression. Patients who have taken corticosteroids in the past may have altered cortisol levels, making nausea and vomiting more difficult to treat. Knowing a patient’s corticosteroid history is critical to developing effective treatment strategies for nausea and vomiting, as previous corticosteroid use can have lasting effects on a patient’s health.
